# Thermo-Magneto-Electric Transport through a Torsion Dislocation in a Type I Weyl Semimetal

**DOI:** 10.3390/nano11112972

**Published:** 2021-11-05

**Authors:** Daniel Bonilla, Enrique Muñoz, Rodrigo Soto-Garrido

**Affiliations:** 1 Physics Institute, Pontificia Universidad Católica de Chile, Avenida Vicuña Mackenna 4860, Santiago 8970117, Chile; dabonilla@uc.cl (D.B.); rodsoto@uc.cl (R.S.-G.); 2 Research Center for Nanotechnology and Advanced Materials, CIEN-UC, Pontificia Universidad Católica de Chile, Avenida Vicuña Mackenna 4860, Santiago 8970117, Chile

**Keywords:** weyl semimetals, transport, torsion, dislocation, magnetic field

## Abstract

Herein, we study electronic and thermoelectric transport in a type I Weyl semimetal nanojunction, with a torsional dislocation defect, in the presence of an external magnetic field parallel to the dislocation axis. The defect is modeled in a cylindrical geometry, as a combination of a gauge field accounting for torsional strain and a delta-potential barrier for the lattice mismatch effect. In the Landauer formalism, we find that due to the combination of strain and magnetic field, the electric current exhibits chiral valley-polarization, and the conductance displays the signature of Landau levels. We also compute the thermal transport coefficients, where a high thermopower and a large figure of merit are predicted for the junction.

## 1. Introduction

Since the experimental discovery of topological insulators, there has been an increasing interest in the search for other materials that may exhibit non-trivial topological properties [1,2,3,4,5]. A remarkable example of three-dimensional gapless topological materials are Weyl semimetals (WSMs). First proposed theoretically [6,7,8,9,10,11,12], WSMs were recently discovered experimentally on TaAs crystals [13] and observed in photonic crystals [14]. In a WSM, the conduction and valence bands touch each other in an even number of points with linear dispersion, referred to as Weyl nodes. These nodes are protected from being gapped because they are monopolar sources of Berry curvature, and thus their charge (chirality) is a topological invariant [12]. In the vicinity of these nodes, low-energy conducting states can be described as Weyl fermions, i.e., massless quasi-particles with pseudo-relativistic Dirac linear dispersion [9,10,11,12,12]. In addition to their intrinsic electronic spin, in Weyl fermions chirality determines the projection of the spin over their momentum direction, a condition often referred to as “spin-momentum locked states”. While Type I WSMs fully respect Lorentz covariance, such condition is not satisfied in Type II WSMs, where the Dirac cones are strongly tilted [4].

The presence of Weyl nodes in the bulk spectrum determines the emergence of Fermi arcs [13], the chiral anomaly, and the chiral magnetic effect, among other remarkable properties [4]. Perhaps the most studied is the chiral anomaly, which is the non-conservation of the independent chiral currents in the presence of non-orthogonal electric and magnetic fields. Therefore, considerable attention has been paid to understand the electronic transport properties of WSMs [15,16,17]. For instance, there are recent works on charge transport [18] in the presence of spin–orbit-coupled impurities [19], electrochemical [20] and nonlinear transport induced by Berry curvature dipoles [21]. Regarding thermoelectric transport in WSMs, it is known that the linear Dirac-type dispersion induces a non-trivial dependence on the chemical potential [22]. Somewhat less explored are the effects of mechanical strain and deformations in WSMs. From the theory perspective, it has been proposed that different sorts of elastic strains can be modeled as gauge fields in WSMs [23,24,25], similar to the case of graphene. In previous works, we have studied the effects of strain and magnetic field on the electronic [26,27] and the thermoelectric [28] transport properties of WSMs, using the Landauer ballistic formalism in combination with the quantum mechanical scattering cross-sections [29]. The study of thermoelectric transport properties is a field of permanent interest, not only regarding WSMs but in a wide range of materials. For instance, there is recent literature involving the experimental determination of the thermoelectric properties (in particular the figure of merit ZT) of Cu-Sn–based thiospinel compounds [30] and SnTe-based materials [31].

**Figure 1:**
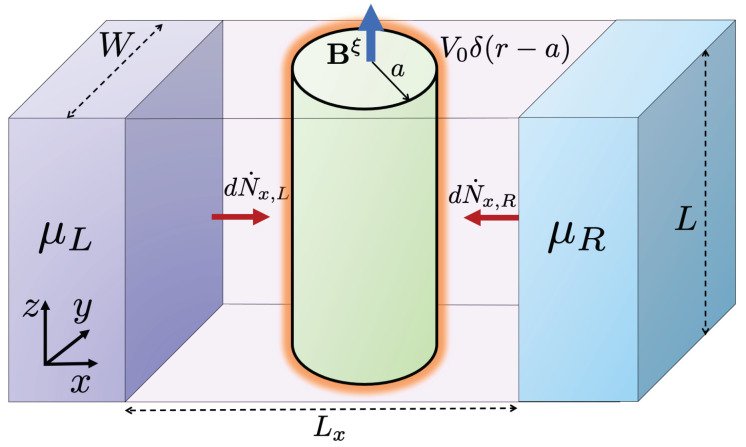
A pictorial description of the system under consideration: A WSM slab of dimensions L×W, with a cylindrical region of radius *a* submitted to a combination of torsional strain and an external magnetic field $B^{\xi }=\left(B+\xi B_{S}\right)\hat{z}$ and an RDSP on the boundary surface of the cylinder.

This work focuses on the effect of a Repulsive Delta-Shell potential (RDSP), in addition to the torsional strain and the external magnetic field studied early on in [26,28], on the thermoelectric transport properties of type I WSMs. The RDSP is a toy model for the surface repulsion produced by the mismatch between the lattices of the strained and the non-strained WSMs. The effect of the delta potential in the context of the Dirac equation is to produce a chiral rotation between the spinors on either side of the boundary that represents the support of the delta function [32,33]. The rotation angle is proportional to the strength of the delta barrier and depends on the chirality of the fermion scattered. This RDSP model for the lattice mismatch of the dislocation is combined with a gauge field representation of the torsional strain in a cylindrical geometry. In addition, an external magnetic field directed along the axis of the dislocation is imposed at the junction, as depicted in Figure 1.

The paper is organized as follows. In Section 2, we establish the Hamiltonian for the model and describe each of its contributions. Then, we proceed with the Landauer formulation for transport accross the junction, first analyzing the sole effect of the RDSP that describes the lattice mismatch, and finally for the full system that includes the torsional strain and the external magnetic field at the WSM junction, with mathematical details presented in the Supplementary Materials. The analysis and discussion of the results are presented in Section 3, with a final summary and conclusions presented in Section 4.

## 2. Theory

As a minimal model for a WSM, we start by considering a free Hamiltonian describing Weyl quasiparticles in the vicinity of each of the nodal points with opposite chirality ξ=±1,

(1) $$\begin{array}{c} H_{\xi }\left(k\right)=v_{F}(\sigma_{1}k_{x}+\sigma_{2}k_{y}+\xi \sigma_{3}k_{z}) \end{array}$$

with $\sigma_{j}$ (j=1,2,3) being the Pauli matrices. The spectrum of this “free” WSM Hamiltonian is given by (for λ=±the band index)

(2) $$\begin{array}{c} E_{\lambda ,k}=\lambda \hbar v_{F}\left|k\right|. \end{array}$$

As depicted in Figure 1, we consider a nanojunction where the WSM is submitted to torsional strain in a cylindrical region of radius *a*, and we further assume that the axial length *L* satisfies L≫a. As discussed in [25], the mechanical strain effect can be incorporated as a gauge field $A_{S}=B_{S}/2(-y{\hat{e}}_{1}+x{\hat{e}}_{2})$, where the constant $B_{S}$ plays the role of a pseudo-magnetic field. Moreover, if a true magnetic field is imposed upon the junction along the axis of the dislocation, i.e., $B={\hat{e}}_{3}B_{0}$, then the combination is described by a node-dependent gauge field $A_{\xi }=B_{\xi }/2(-y{\hat{e}}_{1}+x{\hat{e}}_{2})$, with $B_{\xi }=(B_{0}+\xi B_{S})$ an effective pseudo-magnetic field. In addition to this combined effect, already discussed in our previous work [26,28], here we also consider the lattice mismatch near the boundary of the dislocation. As a simple model for this effect, we include a RDSP potential of the form $V_{RD}\left(r\right)=V_{0}\delta \left(r-a\right)$. Therefore, the quasi-particle states inside the dislocation region correspond to the solutions of the eigenvalue problem

(3) $$\begin{array}{c} {}[H_{\xi }(k+A_{\xi })+V_{RD}\left(r\right)]|\Psi_{n,m}^{\left(\lambda ,\xi \right)}\rangle =E_{\lambda ,n}^{\xi }|\Psi_{n,m}^{\left(\lambda ,\xi \right)}.\rangle \end{array}$$

The spectrum inside the cylindrical region [29] corresponds to relativistic Landau levels with an effective magnetic field $B_{\xi }$ that is node-dependent

(4) $$\begin{array}{c} E_{\lambda ,n}^{\xi }=\lambda \hbar v_{F}\sqrt{2n|B_{\xi }|/{\tilde{\phi }}_{0}+k_{z}^{2}}, \end{array}$$

with ${\tilde{\phi }}_{0}=\left(v_{F}/c\right)\hbar /e$ a modified magnetic flux quantum expressed in terms of the carrier velocity $v_{F}$. The effect of the RDSP potential (see Section S1 of the Supplementary Material for mathematical details) introduces a rotation in the pseudo-spinor components across the dislocation boundary r=a, with an “angle” $\alpha =V_{0}/\left(\hbar v_{F}\right)$

(5) $$\begin{array}{c} {\Psi_{n,m}^{\left(\lambda ,\xi \right)}\left(r\right)|}_{r\to a^{+}}=(\begin{array}{cc} \cos\alpha & -\sin\alpha \\ \sin\alpha & \cos\alpha \end{array}){\Psi_{n,m}^{\left(\lambda ,\xi \right)}\left(r\right)|}_{r\to a^{-}}. \end{array}$$

### 2.1. Transmission and Landauer Conductance

In the Landauer formalism, we define an energy-dependent transmission coefficient along the *x*-direction based on the scattering differential cross section of the junction,

(6) $$\begin{array}{c} \bar{T}\left(E\right)=\int_{-\pi /2}^{\pi /2}d\phi \cos\phi \frac{1}{\sigma (E)}\frac{d\sigma }{d\phi }, \end{array}$$

where σ(E) is the total scattering cross-section at energy *E*. In what follows, we shall assume that the cylindrical dislocation satisfies $L\gg 1/k_{F}$. For instance [34], in TaAs where b∼0.08${\overset{˚}{A}}^{-1}$ and $v_{F}∼1.3\times 10^{5}$ m/s, we have $1/k_{F}∼9$$\overset{˚}{A}$, so even a slab of a few microns is already in the range of validity of this assumption. Moreover, for Cd${}_{3}$As${}_{2}$, b∼0.2${\overset{˚}{A}}^{-1}$ and $v_{F}∼1.5\times 10^{6}$ m/s, $1/k_{F}∼0.8$ $\overset{˚}{A}$ [34], and thus the applicability of this criteria is even more striking in this second example. Therefore, for $L\gg 1/k_{F}$, the differential cross section is given in terms of the scattering phase-shift $\delta_{m}$ for each angular momentum channel *m* [26,29], and integrating over the scattering angle (see Section S2 of the Supplementary Material for mathematical details) we obtain the corresponding total cross section [26,29] $\sigma /L=\frac{4}{k_{⊥}}\sum_{m=-\infty }^{\infty }\sin^{2}\delta_{m}$.

**Figure 2:**
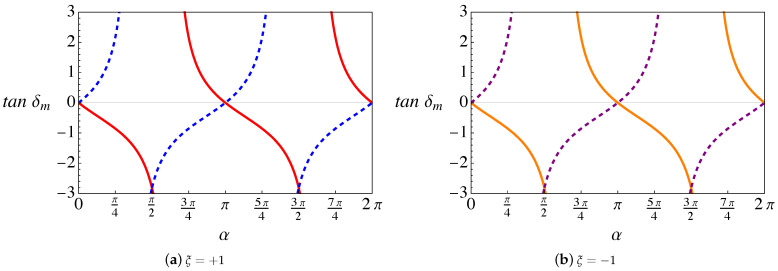
(Color online) Analytical expression for $\tan\delta_{m}$ (in Equation (S29) in Supporting Information) plotted as a function of α. The plots are computed for a wave vector $k_{⊥}∼1/a$ and an orbital angular momentum m=1. (**a**) Node index ξ=1; the red (solid) line corresponds to a band index λ=1 and the blue (dashed) line is for λ=−1. (**b**) Node index ξ=−1; the orange (solid) line corresponds to a band index λ=1 and the purple (dashed) line is for λ=−1.

Let us first consider the effect of the RDSP only. For this case, the current is expressed in terms of the transmission function T(E), evaluated at the free energy eigenvalues $E_{\lambda ,k_{⊥}}$ defined in Equation (2)

(7) $$\begin{array}{c} I=2ev_{F}\sum_{\lambda }\int_{0}^{\infty }dk_{⊥}T\left(E_{\lambda ,k_{⊥}}\right)[f_{L}\left(E_{\lambda ,k_{⊥}}\right)-f_{R}\left(E_{\lambda ,k_{⊥}}\right)], \end{array}$$

where $f_{L/R}\left(E\right)={\left(\exp[\left(E-\mu_{L/R}\right)/\left(k_{B}T_{L/R}\right)]+1\right)}^{-1}$ are the Fermi–Dirac distributions at the chemical potential $\mu_{L/R}$ and temperature $T_{L/R}$ of the left (L) and right (R) metallic contacts, respectively (see Section S3 of the Supplementary Material for mathematical details). The factor of 2 accounts for the (symmetric) contribution from each chiral node ξ=± (see Figure 2). The corresponding expression for the differential conductance $G\left(T,V\right)={\partial I/\partial V|}_{T}$ through the junction is

(8) $$G\left(T,V\right)=2\frac{e^{2}v_{F}}{k_{B}T}\sum_{\lambda }\int_{0}^{\infty }dk_{⊥}T\left(E_{\lambda ,k_{⊥}}\right)f_{L}\left(E_{\lambda ,k_{⊥}}\right)[1-f_{L}\left(E_{\lambda ,k_{⊥}}\right)].$$

Let us now consider the transmission through the junction in its full level of complexity, i.e., including the RDSP for the lattice mismatch, as well as the torsional strain (included via the gauge field model) and the external magnetic field along the axis of the cylindrical dislocation. For this case, scattering is no longer symmetric for each chirality, as seen in the Landau level spectrum $E_{\lambda ,n}^{\xi }$ defined in Equation (4) and in the corresponding scattering phase shift (Figure 2). Therefore, the current for each chirality ξ=± is expressed by the transmission function T(E),

(9) $$\begin{array}{c} I_{\xi }=ev_{F}\sum_{n,\lambda }T\left(E_{\lambda ,n}^{\xi }\right)[f_{L}\left(E_{\lambda ,n}^{\xi }\right)-f_{R}\left(E_{\lambda ,n}^{\xi }\right)], \end{array}$$

with the total current defined by the superposition of both chiral contributions $I=I_{+}+I_{-}$. As before, the differential conductance through the junction is obtained as the voltage derivative of the expression above,

(10) $$\begin{array}{c} G\left(T,V\right)=\frac{e^{2}v_{F}}{k_{B}T}\sum_{\lambda ,n,\xi }T\left(E_{\lambda ,n}^{\xi }\right)f_{L}\left(E_{\lambda ,n}^{\xi }\right)[1-f_{L}\left(E_{\lambda ,n}^{\xi }\right)]. \end{array}$$

### 2.2. Thermoelectric Transport Coefficients

The energy current across the junction arising from each chiral node contribution ξ=± is also expressed in terms of the transmission function T(E) as follows [28]:

(11) $$\begin{array}{c} {\dot{U}}_{\xi }=v_{F}\sum_{n,\lambda }E_{\lambda ,n}^{\xi }T\left(E_{\lambda ,n}^{\xi }\right)[f_{L}\left(E_{\lambda ,n}^{\xi }\right)-f_{R}\left(E_{\lambda ,n}^{\xi }\right)]. \end{array}$$

On the other hand, according to the basic thermodynamic relation TdS=dU−μdN between entropy *S*, internal energy *U*, and particle number *N*, the net heat current transmitted across the junction arising from the node $K_{\xi }$ (for ξ=±) is

(12) $${\dot{Q}}_{\xi }={\dot{U}}_{\xi }-(\mu_{L}{\dot{N}}_{L}^{\xi }-\mu_{R}{\dot{N}}_{R}^{\xi }).$$

The thermal conductance is defined, as usual, under the condition that the net electric current vanishes (I=0)

(13) $$\begin{array}{c} \kappa \left(T,V\right)=-{\frac{\partial \dot{Q}}{\partial \Delta T}|}_{I=0}=-{\frac{\partial \dot{U}}{\partial \Delta T}|}_{I=0}, \end{array}$$

where $\Delta T=T_{R}-T_{L}$ is the temperature difference between the contacts and the total heat flux is given by the superposition from both Weyl nodes $\dot{Q}={\dot{Q}}_{+}+{\dot{Q}}_{-}$, and similar relations hold for the total energy flux $\dot{U}$ and the total electric current *I*. The condition of a vanishing electric current defines an implicit relation between the voltage difference and the thermal gradient across the junction, by I(ΔT,V,T)=0. Therefore, we obtain the Seebeck coefficient by applying the implicit function theorem [28]

(14) $$\begin{array}{c} S\left(T,V\right)=-{\frac{\partial V}{\partial \Delta T}|}_{I=0,T}=\frac{{\frac{\partial I}{\partial \Delta T}|}_{T,V}}{{\frac{\partial I}{\partial V}|}_{T,\Delta T}}, \end{array}$$

where the temperature difference across the junction ΔT(V,T) is obtained as the solution of the equation I(T,V,ΔT)=0. Following the argument above, the thermal conductance defined in Equation (13) is calculated by means of the chain rule and in terms of the Seebeck coefficient [28]

(15) $$\kappa \left(T,V\right)=-{\frac{\partial \dot{U}}{\partial \Delta T}|}_{T,V}+S\left(T,V\right){\frac{\partial \dot{U}}{\partial V}|}_{T,\Delta T}.$$

From the general relations discussed above among the thermoelectric transport coefficients, we obtain the explicit formulae (see Section S4 of the Supplementary Material for mathematical details) for the thermal conductance

(16) $$\begin{array}{ccc} \kappa (T,V) & = & \frac{v_{F}}{k_{B}{\left(T+\Delta T\right)}^{2}}\sum_{\xi ,\lambda ,n}T\left(E_{\lambda ,n}^{\xi }\right)E_{\lambda ,n}^{\xi }[E_{\lambda ,n}^{\xi }-\mu ]f_{R}\left(E_{\lambda ,n}^{\xi }\right)[1-f_{R}\left(E_{\lambda ,n}^{\xi }\right)]\\ & & \;+\;\;S\left(T,V\right)\frac{ev_{F}}{k_{B}T}\sum_{\lambda ,n,\xi }T\left(E_{\lambda ,n}^{\xi }\right)E_{\lambda ,n}^{\xi }\;f_{L}\left(E_{\lambda ,n}^{\xi }\right)[1-f_{L}\left(E_{\lambda ,n}^{\xi }\right)], \end{array}$$

and for the Seebeck coefficient

(17) $$\begin{array}{c} S\left(T,V\right)=-\frac{T\sum_{\lambda ,n,\xi }T\left(E_{\lambda ,n}^{\xi }\right)(E_{\lambda ,n}^{\xi }-\mu )f_{R}\left(E_{\lambda ,n}^{\xi }\right)[1-f_{R}\left(E_{\lambda ,n}^{\xi }\right)]}{e{\left(T+\Delta T\right)}^{2}\sum_{\lambda ,n,\xi }T\left(E_{\lambda ,n}^{\xi }\right)f_{L}\left(E_{\lambda ,n}^{\xi }\right)[1-f_{L}\left(E_{\lambda ,n}^{\xi }\right)]}. \end{array}$$

## 3. Results

In this section, we will apply the analytical results derived in Section 2 to study the response of the transport coefficients to the relevant physical parameters of the model, such as the external magnetic field $B_{0}$, the torsion angle θ, the temperature *T*, and the applied bias voltage *V* [26,28]. In particular, we will analyze the effect of the RDSP, as a model for the lattice mismatch, by varying the $V_{0}$ parameter that characterizes the strength of the repulsive barrier, expressed in terms of the “spinor rotation” angle $\alpha =V_{0}/\hbar v_{F}$.

By considering first the case where only the lattice mismatch effect is present (RDSP) (see Equation (S29) in Section S2 of the Supplementary Material), we notice that the phase shifts depend on the parameter $V_{0}$ through tanα. Therefore, the results depend on α periodically, with period π, as seen in Figure 2. It is also clear (from Equation (S29) in Section S2 of the Supplementary Material) that if the only scattering mechanism is the RDSP, the transmission is maximum for α=nπ, with *n* an integer. At these particular “magic” values, despite the presence of the lattice mismatch, the corresponding interfacial energy barrier becomes transparent to the Weyl fermions of both chiralities ξ=±.

In order to study the additional effect of torsion and magnetic field for TaAs, we estimate [35] $B_{S}\approx 1.8\times 10^{-3}T$ per angular degree of torsion. Furthermore, we have that the modified flux quantum in this material is approximately ${\tilde{\phi }}_{0}\equiv \frac{\hbar v_{F}}{e}=\frac{1}{2\pi }\frac{v_{F}}{c}\frac{hc}{e}=\frac{1}{2\pi }\frac{1.5}{300}\cdot 4.14\times 10^{5}$ T${\overset{˚}{A}}^{2}\approx 330$ T${\overset{˚}{A}}^{2}$. Using these values, we obtain the simple relation between the torsional angle θ (in degrees) and the pseudo-magnetic field $B_{S}$ representing strain

(18) $$B_{S}a^{2}=1.36\;\theta \;{\tilde{\phi }}_{0}.$$

**Figure 3:**
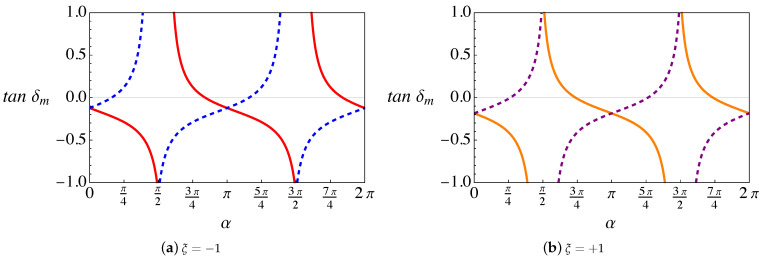
(Color online) Analytical expression for $\tan\delta_{m}$ (in Equation (S32) of Supporting Information) plotted as a function of α. The plots are computed for a quantum number n=1, orbital angular momentum m=1, an external magnetic field $B_{0}a^{2}=25{\tilde{\phi }}_{0}$ and a torsion angle $\theta =10^{\circ }$. (**a**) Node index ξ=1; the red (solid) line corresponds to a band index λ=1 and the blue (dashed) line is for λ=−1. (**b**) Node index ξ=−1; the orange (solid) line corresponds to a band index λ=1 and the purple (dashed) line is for λ=−1.

In this case, the analytical expression for the scattering phase shift is given by Equation (S32) in Section S2 of the Supplementary Material. We notice that the effect of the barrier is again given by tanα, and thus it becomes minimal at “magic” values of α=nπ, i.e., integer multiples of π. However, in this second case, the scattering phase-shift does not vanish, due to the residual combined effect of torsion and magnetic field. This can be seen in Figure 3, where for α=0,π,2π, $\tan\delta_{m}\neq 0$, in contrast to Figure 2. Actually, the value of $\tan\delta_{m}$ for α=nπ (*n* integer) and the consequences of the scattering by the combined magnetic field and torsion, but in the absence of the lattice mismatch barrier, was extensively discussed in our previous works [26,28,29].

Another important aspect to notice is that, when we only consider the lattice mismatch effect, the scattering phase shift is symmetric for both chiral nodes ξ=±1, as seen in Figure 2a,b. In contrast, when the magnetic field and torsion are present, this symmetry is broken, as displayed in Figure 3a,b. As we explained in [26], this occurs because the magnitude of the pseudo-field that combines torsion and magnetic field $B_{\xi }=B_{0}+\xi B_{S}$ depends on the sign of the node chirality, a manifestation of the chiral anomaly which can be also observed in the electric current (see Figure 8a).

### 3.1. Electronic Transport

**Figure 4:**
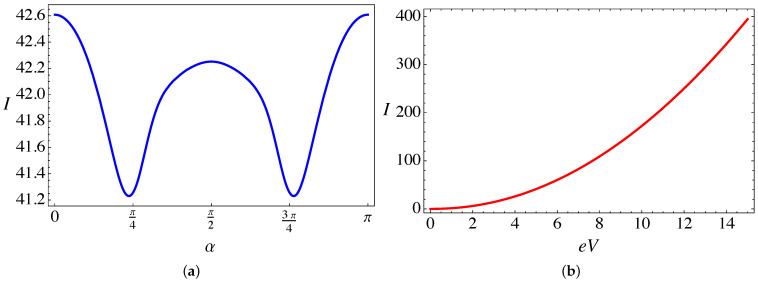
(Color online) Electric current (in units of $ev_{F}/a$) computed from the analytical expression in Equation (7) for the case of the RDSP barrier alone and $T=0.2\;\hbar v_{F}/k_{B}a$: (**a**) Plotted as a function of the applied bias eV (in units of $\hbar v_{F}/a$) and (**b**) plotted as a function of α (dimensionless).

**Figure 5:**
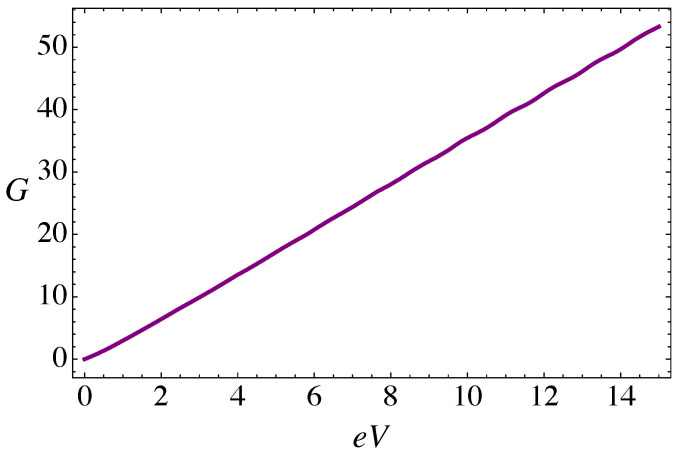
(Color online) Differential conductance (in units of $e^{2}/\hbar$), for the RDSP barrier alone, plotted as function of applied bias eV (in units of $\hbar v_{F}/a$) for α=3π/4 and $T=0.2\;\hbar v_{F}/k_{B}a$, computed from Equation (8).

The electric current (in units of $ev_{F}/a$) is computed from Equation (7) for the case of the RSDP only, in the absence of torsion and magnetic field. Figure 4a shows the periodic dependence of the total current as a function of the dimensionless parameter $\alpha =V_{0}/\left(\hbar v_{F}\right)$ that characterizes the magnitude of the lattice mismatch barrier, and for a temperature $T=0.2\;\hbar v_{F}/k_{B}a$. As expected, the maxima of transmission occur for the “magic angles” α=nπ (*n* integer), and the overall effect of the barrier is to slightly reduce the current, reaching minimal values near α=π/4 and α=3π/4, respectively. The behavior of the current, for the same temperature, as a function of the bias voltage is presented in Figure 4b. As can be seen, for low temperatures the current across the junction displays an approximately quadratic dependence on the applied bias voltage eV (in units of $\hbar v_{F}/a$), that leads to an approximately linear dependence of the differential conductance (in units of $e^{2}/\hbar$) on the bias voltage in Figure 5.

**Figure 6:**
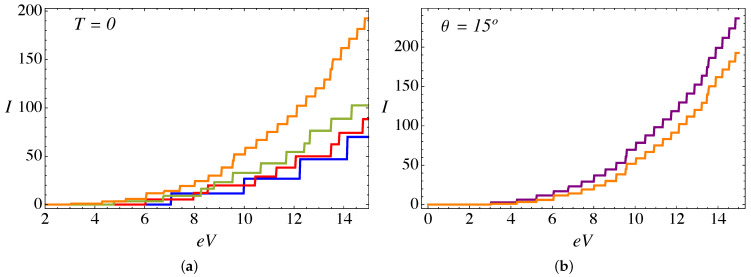
(Color online) (**a**) Electric current (in units of $ev_{F}/a$) as function of applied bias eV (in units of $\hbar v_{F}/a$), computed from the analytical expression in Equation (9) at zero temperature, for an external magnetic field $B_{0}a^{2}=25{\tilde{\phi }}_{0}$ and α=3π/4. The blue line corresponds to a twist angle $\theta =0^{\circ }$, red is for $\theta =5^{\circ }$, green is for $\theta =10^{\circ }$, and the orange line corresponds to $\theta =15^{\circ }$. (**b**) Comparison of electric currents at zero temperature, for $B_{0}a^{2}=25{\tilde{\phi }}_{0}$ and $\theta =15^{\circ }$: the purple line is for α=0, and the orange line corresponds to α=3π/4.

**Figure 7:**
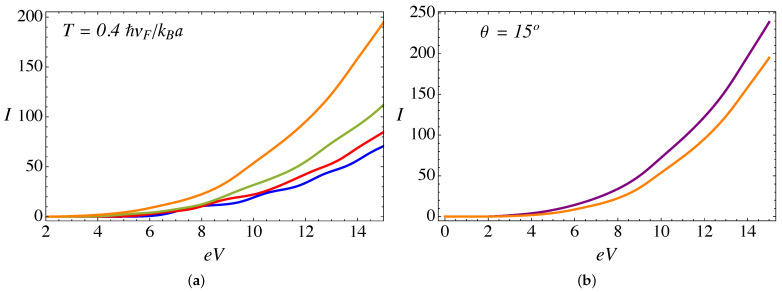
(Color online) (**a**) Electric current (in units of $ev_{F}/a$) plotted as function of applied bias eV (in units of $\hbar v_{F}/a$), computed from the analytical expression in Equation (9) at $T=0.4\;\hbar v_{F}/k_{B}a$, for an external magnetic field $B_{0}a^{2}=25{\tilde{\phi }}_{0}$ and α=3π/4. The blue line corresponds to a twist angle $\theta =0^{\circ }$, red is for $\theta =5^{\circ }$, green is for $\theta =10^{\circ }$, and the orange line corresponds to $\theta =15^{\circ }$. (**b**) Comparison of electric currents at $T=0.4\;\hbar v_{F}/k_{B}a$, for $B_{0}a^{2}=25{\tilde{\phi }}_{0}$ and $\theta =15^{\circ }$: the purple line is for α=0 and orange corresponds to α=3π/4.

**Figure 8:**
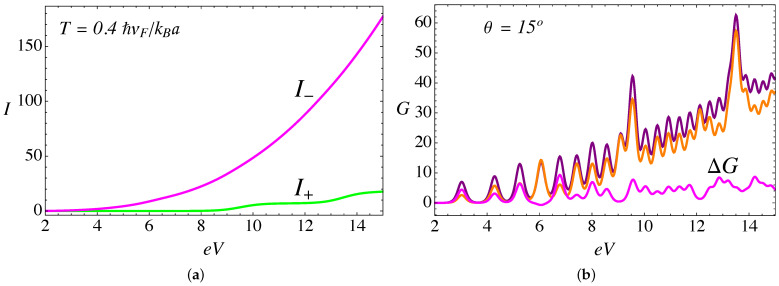
(Color online) (**a**) Node-polarized components of the currents computed for an external magnetic field $B_{0}a^{2}=25{\tilde{\phi }}_{0}$, a torsion angle $\theta =15^{\circ }$, and α=3π/4: the magenta line corresponds to the contribution of $I_{-}$ arising from the $K_{-}$ node and the green line corresponds to the contribution of $I_{+}$ arising from the $K_{+}$ node. (**b**) Comparison of conductance (in units of $e^{2}/\hbar$) as a function of the bias voltage eV (in units of $\hbar v_{F}/a$) for the case of an external magnetic field $B_{0}a^{2}=25{\tilde{\phi }}_{0}$, $T=0.1\hbar v_{F}/k_{B}a$, and a torsion angle $\theta =15^{\circ }$. The purple line is for α=0, orange corresponds to α=3π/4, and the magenta line corresponds to the difference between both ΔG=G(α=0)−G(α=3π/4).

Now, when we include the combined effect of the delta barrier, the external magnetic field, and the torsion strain, the current is calculated from the analytical expression in Equation (9). Figure 6a presents the total current as a function of voltage at zero temperature, an external field $B_{0}a^{2}=25{\tilde{\phi }}_{0}$, a value of α=3π/4, and different values of the torsion angle θ. A remarkable feature at zero temperature is the appearance of plateaus in the current; this is explained by the elastic scattering condition because the incident particle energy must be resonant to one of the pseudo-Landau levels inside the cylinder, and thus each subsequent plateau corresponds to the transmission of an additional Landau level. Such plateaus tend to be smoothed with increasing temperature, as can be seen in Figure 7a. As we discussed in our previous work in the absence of the RDSP contribution [26,28], for a fixed external magnetic field the electric current increases with the torsion angle θ. This effect is due to an enhanced transmission of the Weyl fermions arising from the $K_{-}$ node, as for this particular chirality ξ=−1 the magnitude of the effective pseudo-magnetic field $|B^{-}\left|=\right|B_{0}-B_{S}|$ is smaller, thus increasing the spectral density of pseudo-Landau levels ($∼\sqrt{|B^{\xi }|n}$) for chirality ξ=−1, and consequently an increase in the number of channels available for transmission. Figure 8a presents the difference between the currents originated at each node. Furthermore, for a fixed torsion angle, the transmitted current decreases as the external magnetic field increases [26]. This effect occurs because, by increasing the external field $B_{0}$ (for a fixed torsion field $B_{S}$), the magnitude of the effective pseudo-magnetic field $|B^{\xi }\left|=\right|B_{0}+\xi B_{S}|$ increases for both chiralities ξ=±, thus reducing the density of Landau levels available for transmission. Figure 6b and Figure 7b present a comparison of the current, for α=0 and α=3π/4, at T=0 and $T=0.4\;\hbar v_{F}/k_{B}a$, respectively. We see that the magnitude of the current is reduced while the position of the plateaus remains fixed. This effect is more significant at higher bias voltage, and is due to the repulsive effect of the RDSP barrier that reduces the transmission. Finally, Figure 8b compares the conductance (in units of $e^{2}/\hbar$) as a function of the bias voltage eV (in units of $\hbar v_{F}/a$) for the case of an external magnetic field $B_{0}a^{2}=25{\tilde{\phi }}_{0}$; a torsion angle $\theta =15^{\circ }$; $T=0.1\hbar v_{F}/k_{B}a$; and two different values of the lattice mismatch RDSP barrier, α=0 and α=3π/4, respectively. As expected, the conductance shows peaks as a consequence of the plateaus observed in the current. The effect of the RDSP barrier is to reduce the conductance without affecting the position of the peaks.

### 3.2. Thermal Transport

**Figure 9:**
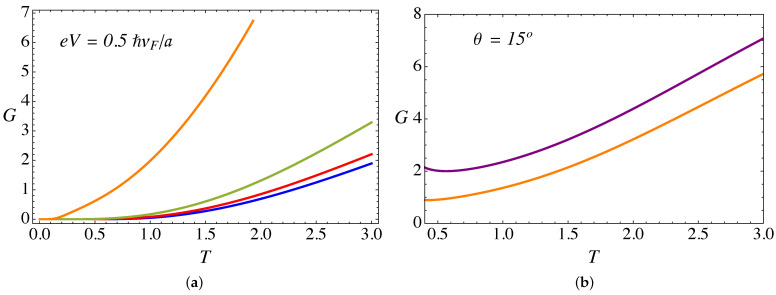
(Color online) (**a**) Conductance (in units of $e^{2}/\hbar$) as a function of temperature (in units of $\hbar v_{F}/k_{B}a$) for external $B_{0}a^{2}=25{\tilde{\phi }}_{0}$, a bias $eV=0.5\;\hbar v_{F}/a$, and α=3π/4. The blue line corresponds to $\theta =0^{\circ }$, red is for $\theta =5^{\circ }$, green is for $\theta =10^{\circ }$, and the orange line corresponds to $\theta =15^{\circ }$. (**b**) Comparison of conductance for $\theta =15^{\circ }$: the purple line is for α=0, whereas the orange line is for α=3π/4.

**Figure 10:**
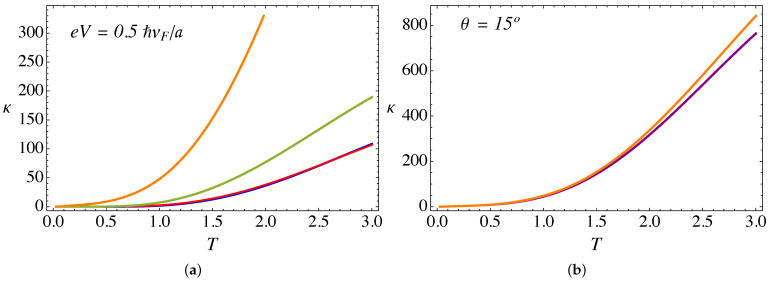
(Color online) (**a**) Thermal conductance (in units of $k_{B}v_{F}/a$) as a function of temperature (in units of $\hbar v_{F}/k_{B}a$), computed from the analytical expression in Equation (16), for external $B_{0}a^{2}=25{\tilde{\phi }}_{0}$, a bias $eV=0.5\;\hbar v_{F}/a$, and α=3π/4. The blue line corresponds to $\theta =0^{\circ }$, red is for $\theta =5^{\circ }$, green is for $\theta =10^{\circ }$, and the orange line corresponds to $\theta =15^{\circ }$. (**b**) Comparison of the thermal conductance for $\theta =15^{\circ }$: the purple line is for α=0 whereas the orange line is for α=3π/4.

Let us now analyze the thermoelectric transport coefficients. Figure 9a presents the electric conductance (in units of $e^{2}/\hbar$) as a function of temperature (in units of $\hbar v_{F}/k_{B}a$) for an external field $B_{0}a^{2}=25{\tilde{\phi }}_{0}$, a bias voltage $eV=0.5\;\hbar v_{F}/a$, α=3π/4, and different torsion angles θ. On the other hand, Figure 10a presents the thermal conductance (in units of $e^{2}/\hbar$) as a function of temperature, for the same set of parameters as in Figure 9a. Both transport coefficients show a monotonic increase with temperature. This effect occurs because Weyl fermions are the same entities transporting current and energy, because as we explained in Section 2, in the present work we only consider the electronic contribution to the transport. Other effects, such as phonons, will be analyzed in future work.

From Figure 9a and Figure 10a, it is clear that both transport coefficients, i.e., the thermal and the electric conductance, increase with torsion. This effect, already observed in our previous work in the absence of the lattice mismatch barrier contribution [28], is due to the enhancement of the pseudo-Landau levels density of states arising from the ξ=−1 chiral node, as already discussed in the previous section.

**Figure 11:**
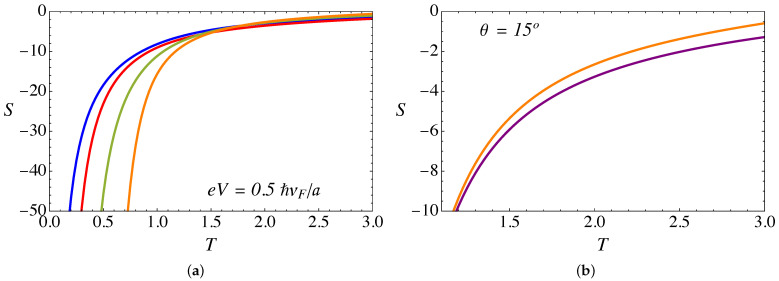
(Color online) (**a**) Seebeck coefficient (in units of $k_{B}/e$) computed from the analytical expression in Equation (17) as a function of temperature *T* (in units of $\hbar v_{F}/k_{B}a$). The plot is for fixed $B_{0}a^{2}=25{\tilde{\phi }}_{0}$, a bias $eV=0.5\;\hbar v_{F}/a$, and α=3π/4. The blue line corresponds to $\theta =0^{\circ }$, red is for $\theta =5^{\circ }$, green is for $\theta =10^{\circ }$ and the orange line corresponds to $\theta =15^{\circ }$. (**b**) Comparison of the Seebeck coefficient for $\theta =15^{\circ }$: the purple line is for α=0 whereas the orange line is for α=3π/4.

Figure 11a shows the Seebeck coefficient (in units of $k_{B}/e$) as a function of temperature (in units of $\hbar v_{F}/k_{B}a$), for the same set of parameters as in Figure 9a and Figure 10a. We have chosen the chemical potential as $\mu =1.0\;\hbar v_{F}/a>0$, such that the negative charge carriers dominate the transport, which explains the negative sign of the Seebeck coefficient. As can be seen, the slope of *S* is very steep at low temperatures and varies monotonically. On the other hand, the absolute value of *S* increases with the torsion angle θ.

Now, let us discuss the effect of the RDSP barrier representing the lattice mismatch via the parameter α. Figure 9b and Figure 10b present a comparison of the α=0 and α=3π/4 cases for the electric and thermal conductance, respectively, as a function of temperature. For the case of electric conductance, the effect is hardly noticeable, with a tiny decrease of the conductance for the case with the RDSP barrier present, α≠0. On the contrary, the effect is most notorious for the case of the thermal conductance, which increases when the lattice mismatch barrier is present. In both cases, the effect tends to be more significant at high temperatures.

For the characterization of the thermoelectric performance of this WSM junction, a useful quantity is the magnitude of the figure of merit ZT, defined by the well-known formula

(19) $$ZT=S^{2}\frac{T\;G(T,V)}{\kappa (T,V)}.$$

**Figure 12:**
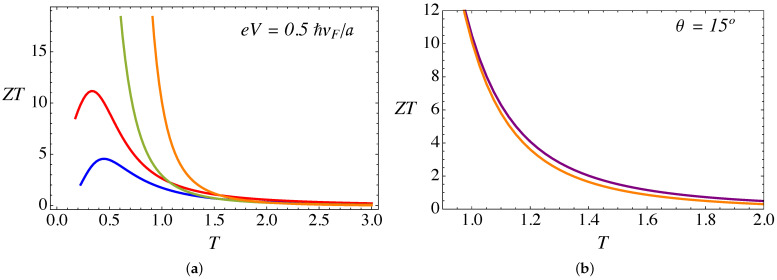
(Color online) (**a**) The figure of merit ZT (dimensionless) as a function of temperature (in units of $\hbar \;v_{F}/k_{B}a$), calculated for fixed $B_{0}a^{2}=25{\tilde{\phi }}_{0}$, a bias $eV=0.5\;\hbar v_{F}/a$, and α=3π/4. The blue line corresponds to $\theta =0^{\circ }$, red is for $\theta =5^{\circ }$, green is for $\theta =10^{\circ }$, and the orange line corresponds to $\theta =15^{\circ }$. (**b**) Comparison of the figure of merit ZT for $\theta =15^{\circ }$: the purple line is for α=0 whereas the orange line is for α=3π/4.

Figure 12a presents the figure of merit ZT (dimensionless), as a function of temperature and for various torsion angles θ. As we showed in our previous work, in the absence of the lattice mismatch effect [28], it is important to notice that extremely high values of ZT can be achieved through the combination of external magnetic field and torsional strain. The value of ZT increases with the torsion angle θ, and the effect is more appreciable at low temperatures. The effect of the RDSP barrier representing lattice mismatch by the parameter α is shown in Figure 12b. The presence of the barrier produces a small reduction of the figure of merit at high temperatures.

It is also pertinent to explore the deviation from the metallic behavior by studying the Lorenz number as a function of temperature. The Lorenz number is defined by the formula

(20) $$L=\frac{\kappa (T,V)}{TG(T,V)}.$$

**Figure 13:**
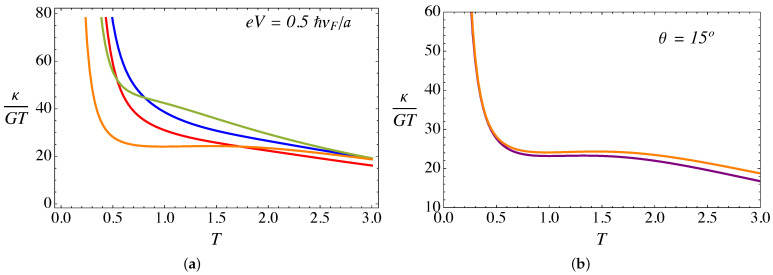
(Color online) (**a**) The Lorenz number (in units of $k_{B}^{2}/e^{2}$) as a function of temperature (in units of $\hbar \;v_{F}/k_{B}a$), calculated for fixed $B_{0}a^{2}=25{\tilde{\phi }}_{0}$, a bias $eV=0.5\;\hbar v_{F}/a$ and α=3π/4. The blue line corresponds to $\theta =0^{\circ }$, red is for $\theta =5^{\circ }$, green is for $\theta =10^{\circ }$, and the orange line corresponds to $\theta =15^{\circ }$. (**b**) Comparison of the Lorenz number for $\theta =15^{\circ }$: the purple line is for α=0 whereas the orange line is for α=3π/4.

The Lorenz number is represented, at fixed bias and magnetic field, as a function of temperature for different values of torsion in Figure 13a. Strong deviations from the Wiedemann–Franz law are observed at low temperatures. This effect occurs because the electronic conductance exhibits a non-metallic behavior at low temperatures, due to the discrete pseudo-Landau level spectrum, as can be seen in the staircase pattern in Figure 6. It is precisely this effect that explains the extremely high ZT values at low temperatures, in agreement with the experimental evidence reported [36] that suggested values as high as ZT∼10. In contrast with the ZT behavior, the presence of the delta barrier increasing the Lorenz number at high temperatures, as can be seen in Figure 13b. This trend is explained, as we discussed previously, by the fact that at high temperatures the thermal conductance increases with the delta barrier, while the electric conductance slightly decreases.

## 4. Discussion

In this work, we studied the thermoelectric transport properties of a type I Weyl semimetal with a torsional defect, in the presence of an external magnetic field along the axis of the dislocation in a cylindrical geometry. Moreover, the effect of torsion was modeled by a combination of a gauge field representation, and a repulsive delta-shell potential (RDSP) representing the lattice mismatch at the edge of the cylindrical region. We remark that the mechanical gauge field, in combination with the external magnetic field imposed upon the region, combine into an effective node-dependent pseudo-magnetic field $B^{\xi }=B+\xi B_{S}$ (for ξ=±) that breaks time-reversal symmetry and thus the nodal symmetry. Therefore, our analysis shows that the electronic states within the region correspond to effective node-polarized Landau levels, leading to a node-polarization effect of the total electric current $I=I_{+}+I_{-}$. In particular, the low-temperature differential conductance displays the corresponding characteristic trend of discrete peaks corresponding to each of such Landau levels. We also demonstrated that the effect of the lattice-mismatch, represented by the RDSP, is periodic in the strength of the repulsive barrier $V_{0}$, in the form $\tan(V_{0}/\hbar v_{F})$, thus revealing the presence of “magic angles” (the zeroes of the tangent) where the barrier becomes transparent. This somewhat surprising effect is a manifestation of the Klein tunneling effect of Dirac’s theory, observed in this particular context and geometry. Finally, we also studied the thermoelectric transport coefficients—thermal conductivity and Seebeck—as a function of temperature, external magnetic field, torsion, and strength of the lattice mismatch (RDSP).

We would like to emphasize that our analytical equations, and the corresponding figures presented in Section 3, are expressed in terms of dimensionless groups involving structural parameters (such as the radius *a* of the torsional defect and the dimensions *W* and *L* of the WSM slab) as well as the material’s specific parameters (such as the Fermi velocity $v_{F}$). This has the advantage that the equations presented are quite general, and thus our theoretical predictions for the transport coefficients can be compared with specific experimental measurements by choosing the appropriate material-dependent parameters. For instance, choosing the dimensions of the slab as W∼L∼50nm and the radius of the cylindrical strip as a∼15nm, we obtain an electrical resistivity $\rho ∼2.15\times 10^{-4}\;\;\Omega m$ which is within the range reported in [37] ($\rho ∼2\times 10^{-2}\;\;\Omega m$ for Bi and $\rho ∼10^{-5}\;\;\Omega m$ for TaP). On the other hand, for the case of the thermal conductivity, using the Fermi velocity $v_{F}∼1.5\times 10^{6}\;\;m/s$ for the material Cd${}_{3}$As${}_{2}$ [34], and the same values for *a*, *L*, and *W* as before, we found a value of κ∼6.6W/mK which is of the same order of magnitude to those reported in [36] (∼3W/mK for Pb${}_{1-x}$Sn${}_{x}$Se) and in [37] (∼5−25W/mK for TaP).

Finally, we point out that our theoretical calculations suggest that a very high figure of merit can be obtained from such configuration (torsional strain + RDSP), thus constituting a very interesting candidate for thermoelectric applications in energy harvesting.

## Data Availability

Data is contained within the article or supplementary material.

## Supplementary Materials

The following are available online at https://www.mdpi.com/article/10.3390/nano11112972/s1.

## Abbreviations

The following abbreviations are used in this manuscript:

WSM	Weyl semimetal
RDSP	Repulsive delta-shell potential
