# Supplementary material for: Thermo-Magneto-Electric Transport through a Torsion Dislocation in a Type I Weyl Semimetal
Source: Nanomaterials (Basel). 2021 Nov 5;11(11):2972. doi: 10.3390/nano11112972 (PMC8619483; doi:10.3390/nano11112972)
Supplement: Supplementary file 1 [file nanomaterials-11-02972-s001.zip › nanomaterials-1430559-supplementary.pdf]

Supplementary Material for

"Thermo-magneto-electric transport through a torsion dislocation in a type I Weyl Semimetal"

Daniel Bonilla [1], Enrique Muñoz [1,2,*] and Rodrigo Soto-Garrido [1]

[1] *Physics Institute, Pontificia Universidad Católica de Chile, Avenida Vicuña Mackenna 4860, Santiago, Chile.*

[2] *Research Center for Nanotechnology and Advanced Materials, CIEN-UC, Pontificia Universidad Católica de Chile, Avenida Vicuña Mackenna 4860, Santiago, Chile.*

[*] Correspondence: munozt@fis.puc.cl; Tel.: +56-2-2354-7625

1 Solution of the eigenvalue problem for the RDSP

We start with the eigenvalue problem

$$\left[H_{\xi}\left(\mathbf{k}+\mathbf{A}_{\xi}\right)+V_{RD}(r)\right]|\Psi_{n,m}^{(\lambda,\xi)}\rangle = E_{\lambda,n}^{\xi}|\Psi_{n,m}^{(\lambda,\xi)}\rangle, \tag{1}$$

and represent it in the cylindrical coordinate basis (r,ϕ,z). In this representation we have $\mathbf{k}=-i\nabla$, $V_{RD}(r)=V_0\delta(r-a)$, and we can choose the vector potential in the gauge $\mathbf{A}_{\xi}=A_{\phi}(r)\hat{\phi}$, with $A_{\phi}(r)=B_{\xi}r/2$ for $r<a$ and $A_{\phi}(r)=0$ for $r>a$. Therefore, we obtain

$$\left[\xi v_F\left\{\left(\hat{r}\cdot\boldsymbol{\sigma}\right)\left[\frac{\hbar}{i}\left(\frac{\partial}{\partial r}+\frac{1}{2r}\right)+i\sigma_3\left(\frac{\hat{J}_3}{r}-eA_{\phi}(r)\right)\right]-i\hbar\sigma_3\frac{\partial}{\partial z}\right\}+V_0\delta(r-a)\right]\Psi_{n,m}^{(\lambda,\xi)}(\mathbf{r})=E_{\lambda,n}^{\xi}\Psi_{n,m}^{(\lambda,\xi)}(\mathbf{r}), \tag{2}$$

where the total angular momentum is $\hat{J}_3=\hat{L}_3+\frac{\hbar}{2}\sigma_3$, the orbital angular momentum is $\hat{L}_3=-i\partial_{\phi}$, and we have used that $\hat{\phi}\cdot\boldsymbol{\sigma}=i\left(\hat{r}\cdot\boldsymbol{\sigma}\right)\sigma_3$, with

$$\hat{r}\cdot\boldsymbol{\sigma}=\begin{pmatrix}0 & e^{-i\phi}\\ e^{i\phi} & 0\end{pmatrix}. \tag{3}$$

Because of the cylindrical symmetry of the system, the Hamiltonian has no explicit dependence on the variable z, and hence it is possible to decompose the total solution in angular momentum channels, such that the pseudo-spinors can be written in the form

$$\Psi_{n,m}^{(\lambda,\xi)}(\mathbf{r}) = r^{-\frac{1}{2}} \begin{bmatrix} f_m(r)e^{im\phi} \\ -i\, g_m(r)e^{i(m+1)\phi} \end{bmatrix} e^{k_z z}. \tag{4}$$

It is straightforward to check that the pseudo-spinors in Eq.(4) are eigenstates of the total angular momentum, i.e., $\hat{J}_3\Psi_{n,m}^{(\lambda,\xi)}(\mathbf{r}) = m_j\hbar\Psi_{n,m}^{(\lambda,\xi)}(\mathbf{r})$ with $m_j = \pm\frac{1}{2}, \pm\frac{3}{2}, \cdots$ and the orbital angular momentum quantum number is $m = m_j - 1/2$. Insertion of the pseudo-spinor in Eq. (4) in the eigenvalue problem Eq. (2) leads to the radial equation

$$\begin{pmatrix} k_z + \frac{\xi V_0}{\hbar v_F}\delta(r-a) & -\frac{d}{dr} - \left(\frac{m+1/2}{r} - \frac{e}{\hbar}A_\phi\right) \\ \frac{d}{dr} - \left(\frac{m+1/2}{r} - \frac{e}{\hbar}A_\phi\right) & -k_z + \frac{\xi V_0}{\hbar v_F}\delta(r-a) \end{pmatrix} \begin{bmatrix} f_m(r) \\ g_m(r) \end{bmatrix} = \frac{\xi E_{\lambda,n}^{\xi}}{\hbar v_F} \begin{bmatrix} f_m(r) \\ g_m(r) \end{bmatrix}. \tag{5}$$

Let us introduce the new quantities

$$\alpha \equiv \frac{\xi V_0}{\hbar v_F}, \quad \varepsilon \equiv \frac{\xi E_{\lambda,n}^{\xi}}{\hbar v_F}, \quad \Phi_m(r) \equiv \begin{bmatrix} f_m(r) \\ g_m(r) \end{bmatrix}. \tag{6}$$

Then, it is direct to notice that Eq.(5) can be cast in the form

$$\frac{d}{dr}\Phi_m(r) + \left[\mathbf{M}(r) + i\alpha\delta(r-a)\sigma_2\right]\Phi_m(r) = 0, \tag{7}$$

where $\mathbf{M}(r)$ is a 2×2 matrix given by

$$\mathbf{M}(r) = \begin{pmatrix} -\left(\frac{m+1/2}{r} - \frac{e}{\hbar}A_\phi\right) & -k_z - \varepsilon \\ -k_z + \varepsilon & \left(\frac{m+1/2}{r} - \frac{e}{\hbar}A_\phi\right) \end{pmatrix}. \tag{8}$$

We notice that Eq. (7) can be expressed as a total derivative as follows

$$\frac{d}{dr}\left\{e^{\int_c^r d\rho[\mathbf{M}(\rho)+i\alpha\delta(\rho-a)\sigma_2]}\Phi_m(r)\right\} = 0, \tag{9}$$

where $0 < c < a$ is a constant. Integrating Eq. (9) from $r = a - \epsilon$ to $r = a + \epsilon$, with ϵ a positive infinitesimal, we have

$$e^{\int_{a-\epsilon}^{a+\epsilon} d\rho\mathbf{M}(\rho)+i\alpha\sigma_2}\ \Phi_m(r)\big|_{r=a+\epsilon} = \Phi_m(r)\big|_{r=a-\epsilon}. \tag{10}$$

Now, taking the limit $\epsilon \to 0$ we obtain the matching condition at the boundary of the cylinder

$$e^{i\alpha\sigma_2}\,\Phi_m(r)|_{r\to a^+} = \Phi_m(r)|_{r\to a^-}\,, \tag{11}$$

provided that according to Eq. (8), the matrix $\mathbf{M}(r)$ is a regular function across of $r = a$. Using the well known result for $e^{i\alpha\sigma_2}$ and the complete form of the pseudo-spinor in Eq. (4), the boundary condition can be written in closed form as

$$\Psi^{(\lambda,\xi)}_{n,m}(\mathbf{r})\Big|_{r\to a^+} = \begin{pmatrix} \cos\alpha & -\sin\alpha \\ \sin\alpha & \cos\alpha \end{pmatrix} \Psi^{(\lambda,\xi)}_{n,m}(\mathbf{r})\Big|_{r\to a^-}\,, \tag{12}$$

where $\Psi^{(\lambda,\xi)}_{n,m}(\mathbf{r})\Big|_{r\to a^+}$ is the solution outside the cylinder and $\Psi^{(\lambda,\xi)}_{n,m}(\mathbf{r})\Big|_{r\to a^-}$ is the solution inside the cylinder.

2 Scattering analysis

In this section we perform the standard scattering analysis using the method of partial waves[1]. We closely follow the treatment of Refs. [2], [3] and consider the elastic scattering for an incident 'free' pseudo-spinor with momentum $\hbar\mathbf{k}$ (where $\mathbf{k} = (\mathbf{k}_\perp, k_z)$ and $\mathbf{k}_\perp = (k_x, k_y)$) and energy $E_{\lambda,k} = \lambda\hbar v_F|\mathbf{k}|$. The incident 'free' fermion is the solution of the eigenvalue equation $H_\xi(\mathbf{k})\Psi^{(\lambda,\xi)}_{\mathrm{inc},k}(\mathbf{r}) = \lambda\hbar v_F|\mathbf{k}|\Psi^{(\lambda,\xi)}_{\mathrm{inc},k}(\mathbf{r})$, i.e.,

$$\Psi^{(\lambda,\xi)}_{\mathrm{inc},k}(\mathbf{r}) = \frac{1}{\sqrt{1+\beta^2}}\begin{bmatrix} 1 \\ \beta \end{bmatrix} e^{ik_\perp r\cos\phi + ik_z z}, \tag{13}$$

where we have introduced the dimensionless constant

$$\beta \equiv \frac{\lambda\xi|\mathbf{k}| - k_z}{k_\perp} \equiv \frac{k_\perp}{k_z + \xi\lambda|\mathbf{k}|}. \tag{14}$$

Outside the cylindrical strip the particles are free, so we make the substitution $V_0 = 0$, $A_\phi = 0$ and $E^{\xi}_{\lambda,n} = \lambda\hbar v_F|\mathbf{k}|$ in the radial equation Eq.(5) to obtain

$$\begin{pmatrix} k_z & \hat{D}_0^\dagger \\ \hat{D}_0 & -k_z \end{pmatrix}\begin{bmatrix} f_m(r) \\ g_m(r) \end{bmatrix} = \lambda\xi|\mathbf{k}|\begin{bmatrix} f_m(r) \\ g_m(r) \end{bmatrix}, \tag{15}$$

where $\hat{D}_0 = \frac{d}{dr} - \frac{m+1/2}{r}$ and $\hat{D}_0^\dagger = -\frac{d}{dr} - \frac{m+1/2}{r}$. The radial components of the spinor in the Eq.(15) are obtained solving the diagonal system

$$\begin{bmatrix} \hat{D}_0^\dagger \hat{D}_0 + k_z^2 - |\mathbf{k}|^2 & 0 \\ 0 & \hat{D}_0 \hat{D}_0^\dagger + k_z^2 - |\mathbf{k}|^2 \end{bmatrix} \begin{bmatrix} f_m(r) \\ g_m(r) \end{bmatrix} = 0, \tag{16}$$

where

$$\hat{D}_0^\dagger \hat{D}_0 = -\frac{d^2}{dr^2} + \frac{(m+1/2)(m-1/2)}{r^2}, \tag{17}$$

$$\hat{D}_0 \hat{D}_0^\dagger = -\frac{d^2}{dr^2} + \frac{(m+1/2)(m+3/2)}{r^2}. \tag{18}$$

We then have the pair of differential equations

$$\begin{aligned} \left[-\frac{d^2}{dr^2} + \frac{(m+1/2)(m-1/2)}{r^2} - k_\perp^2 \right] f_{m_j}(r) &= 0, \\ \left[-\frac{d^2}{dr^2} + \frac{(m+1/2)(m+3/2)}{r^2} - k_\perp^2 \right] g_{m_j}(r) &= 0. \end{aligned} \tag{19}$$

The general solution for the system of differential equations in Eq. (19) is given in terms of the Bessel functions of the first and second kind

$$\begin{aligned} f_m(r) &= c_1 \sqrt{k_\perp r} J_m(k_\perp r) + c_2 \sqrt{k_\perp r} Y_m(k_\perp r), \\ g_m(r) &= c_3 \sqrt{k_\perp r} J_{m+1}(k_\perp r) + c_4 \sqrt{k_\perp r} Y_{m+1}(k_\perp r). \end{aligned} \tag{20}$$

However, the radial equation in Eq. (15) imposes a relation between the upper and lower components, i.e.,

$$g_m(r) = \frac{-k_\perp}{k_z + \xi\lambda|\mathbf{k}|} \left[c_1 \sqrt{k_\perp r} J_{m+1}(k_\perp r) + c_2 \sqrt{k_\perp r} Y_{m+1}(k_\perp r) \right], \tag{21}$$

where we have applied the identity [4]

$$\frac{dZ_\alpha}{dx} - \frac{\alpha}{x} Z_\alpha(x) = -Z_{\alpha+1}(x). \tag{22}$$

Equating Eq. (21) with the last of Eq. (20) we have that

$$c_3 = -\beta c_1, \quad c_4 = -\beta c_2, \tag{23}$$

where β is given in Eq. (14).

Phase Shift

In order to find the phase shift δ_m for each angular momentum channel m, let us first consider the asymptotic form of the Bessel functions for large[4] r

$$\begin{aligned} J_m(k_\perp r) &\sim \sqrt{\frac{2}{\pi k_\perp r}} \cos\left(k_\perp r - \left(m + \frac{1}{2}\right)\frac{\pi}{2}\right), \\ Y_m(k_\perp r) &\sim \sqrt{\frac{2}{\pi k_\perp r}} \sin\left(k_\perp r - \left(m + \frac{1}{2}\right)\frac{\pi}{2}\right). \end{aligned} \tag{24}$$

Taking these forms into account, we have that the general 'free' spinor solution outside of the cylinder, for large r, has the asymptotic form

$$\Psi^{(\lambda,\xi)}_{\text{out},k}(\mathbf{r}) \sim \sqrt{\frac{2}{\pi k_\perp r}} C_m \begin{bmatrix} e^{im\phi} \cos\left(k_\perp r - \left(m + \frac{1}{2}\right)\frac{\pi}{2} + \delta_m\right) \\ i\beta\, e^{i(m+1)\phi} \cos\left(k_\perp r - \left(m + \frac{3}{2}\right)\frac{\pi}{2} + \delta_m\right) \end{bmatrix} e^{ik_z z}. \tag{25}$$

Here, we have defined the global coefficients and phase shifts by

$$\begin{aligned} C_m =& \sqrt{k_\perp}\sqrt{c_1^2 + c_2^2}, \\ \tan\delta_m =& -\frac{c_2}{c_1}. \end{aligned} \tag{26}$$

If the only mechanism responsible for scattering is the RDSP, then we have free pseudo-spinors in both regions, inside and outside the cylinder. The solution for the radial components of the spinor in the exterior region ($r > a$) appears in Eq. (20), with the condition in Eq. (23). Then, on the one hand we have

$$\begin{bmatrix} f_m(r) \\ g_m(r) \end{bmatrix}_{r\to a^+} = \sqrt{k_\perp a} \begin{bmatrix} c_1 J_m(k_\perp a) + c_2 Y_m(k_\perp a) \\ -\beta c_1 J_m(k_\perp a) - \beta c_2 Y_m(k_\perp a) \end{bmatrix}. \tag{27}$$

On the other hand, the well behaved solution inside the cylinder ($r < a$) is

$$\begin{bmatrix} f_m(r) \\ g_m(r) \end{bmatrix}_{r\to a^-} = \tilde{C}\sqrt{k_\perp a} \begin{bmatrix} J_m(k_\perp a) \\ -\beta J_m(k_\perp a) \end{bmatrix}, \tag{28}$$

where $\tilde{C}$ is an arbitrary constant. Now, applying the matching condition in Eq. (12) at the boundary of the cylinder ($r = a$) to the solutions in Eq. (27) and Eq. (28), we have a system of equations that determines the constants c_1 and c_2 in terms of the arbitrary constant $\tilde{C}$. Then, from the definition of the phase shift in

Eq. (26) we obtain

$$\tan\delta_m(\alpha) = \frac{\tan\alpha\left[J_m^2 + \beta^2 J_{m+1}^2\right]}{\tan\alpha\left[J_m\cdot Y_m + \beta^2 J_{m+1}\cdot Y_{m+1}\right] - \beta\left[J_{m+1}\cdot Y_m - J_m\cdot Y_{m+1}\right]}, \tag{29}$$

where α is given in the Eq. (6), β is defined in the Eq. (14) and it is understood that all the Bessel functions, J_m and Y_m, have argument $k_\perp a$. We can notice that when $\alpha = 0$, i.e., there is no scattering mechanism, the expression in Eq. (29) reduces to $\tan\delta_m = 0$. As expected, when there is no scattering, there is no phase shift.

Now, we consider the case in which, inside the cylinder, there is an external magnetic field, a torsion pseudo-field, and the RDSP. The interior solution appears in the appendix of Ref.[2]. If we introduce the dimensionless parameter $z_a = |B_\xi|a^2/2\tilde{\phi}_0$, the solution inside the cylinder (for $n > 0$) can be written as

$$\Psi_{n,m}^{(\lambda,\xi)}(\mathbf{r})\Big|_{r\to a^-} = C_{n,m}^{\xi,\lambda}\begin{bmatrix} z_a^{\frac{|m|}{2}}\, e^{-\frac{1}{2}z_a} L_{n_\rho}^{|m|}(z_a)\, e^{im\phi} \\ i\,\varrho_n^\xi z_a^{\frac{|m+1|}{2}} e^{-\frac{1}{2}z_a} L_{n'_\rho}^{|m+1|}(z_a)\, e^{i(m+1)\phi}\end{bmatrix} e^{ik_z z}, \tag{30}$$

where the coefficients are defined by

$$\begin{aligned} n_\rho &= n - \theta(-B_\xi) - \frac{|m| - m\,\mathrm{sign}B_\xi}{2}, \\ n'_\rho &= n_\rho - \theta(B_\xi) + \theta(-m), \\ \varrho_n^\xi &= \frac{\sqrt{2|B_\xi|/\tilde{\phi}_0}\; n^{\theta(-m)}}{\lambda\xi\sqrt{2n|B_\xi|/\tilde{\phi}_0 + k_z^2} + k_z}. \end{aligned} \tag{31}$$

Here, $\theta(x)$ is the Heaviside step function and the value of m is restricted by the principal quantum number n as follows: for $\mathrm{sign}B_\xi = +1$, we have $-n \le m < +\infty$, and for $\mathrm{sign}B_\xi = -1$, we have $-\infty < m \le n-1$. Proceeding as before, we apply the matching condition in Eq. (12) (at $r = a$) to the exterior solution in Eq. (27) and interior solution in Eq. (30), in order to find the constants c_1 and c_2 in terms of $C_{n,m}^{\xi,\lambda}$. The corresponding phase shift is computed using Eq.(26), and we finally get

$$\tan\delta_m(\alpha, B^\xi) = \frac{\beta J_{m+1} - \varrho_n^\xi J_m\cdot z_a^{\frac{|m+1|-|m|}{2}}\dfrac{L_{n'_\rho}^{|m+1|}(z_a)}{L_{n_\rho}^{|m|}(z_a)} + \tan\alpha\left[J_m + \beta\varrho_n^\xi J_{m+1}\cdot z_a^{\frac{|m+1|-|m|}{2}}\dfrac{L_{n'_\rho}^{|m+1|}(z_a)}{L_{n_\rho}^{|m|}(z_a)}\right]}{\beta Y_{m+1} - \varrho_n^\xi Y_m\cdot z_a^{\frac{|m+1|-|m|}{2}}\dfrac{L_{n'_\rho}^{|m+1|}(z_a)}{L_{n_\rho}^{|m|}(z_a)} + \tan\alpha\left[Y_m + \beta\varrho_n^\xi Y_{m+1}\cdot z_a^{\frac{|m+1|-|m|}{2}}\dfrac{L_{n'_\rho}^{|m+1|}(z_a)}{L_{n_\rho}^{|m|}(z_a)}\right]}, \tag{32}$$

where, as before, all the Bessel functions are evaluated at $k_\perp a$. It is straightforward to show that for the case

$\alpha = 0$, the expression for the phase shift reduces to

$$\tan\delta_m(B^\xi) = \frac{\beta J_{m+1}(k_\perp a) - \varrho_n^\xi J_m(k_\perp a) z_a^{\frac{|m+1|-|m|}{2}} \dfrac{L_{n'_\rho}^{|m+1|}(z_a)}{L_{n_\rho}^{|m|}(z_a)}}{\beta Y_{m+1}(k_\perp a) - \varrho_n^\xi Y_m(k_\perp a) z_a^{\frac{|m+1|-|m|}{2}} \dfrac{L_{n'_\rho}^{|m+1|}(z_a)}{L_{n_\rho}^{|m|}(z_a)}}, \tag{33}$$

which is the phase shift for the case when the external magnetic field and the torsion field are present inside the cylinder, but there is no RDSP at the boundary [2], [3].

Scattering Amplitudes

Outside the cylindrical strip, in the limit $r \gg a$, the 'free' state is a linear combination of the incident and the scattered spinors

$$\Psi^{(\lambda,\xi)}_{\text{out},k}(\mathbf{r})) \sim \frac{1}{\sqrt{1+\beta^2}} \begin{bmatrix} 1 \\ \beta \end{bmatrix} e^{ik_\perp r\cos\phi + ik_z z} + \begin{bmatrix} f_1(\phi) \\ f_2(\phi) \end{bmatrix} \frac{e^{ik_\perp r + ik_z z}}{\sqrt{r}}. \tag{34}$$

Here, $f_1(\phi)$ and $f_1(\phi)$ are the scattering amplitudes for each component of the spinor. We want to study the contribution of each partial wave of orbital angular momentum $m\hbar$. For this purpose, we use the following expansion of plane waves in terms of Bessel functions [4]

$$e^{ikr\cos\phi} = \sum_{m=-\infty}^{\infty} i^m e^{im\phi} J_m(kr) \sim \sqrt{\frac{2}{\pi k r}} \sum_{m=-\infty}^{\infty} i^m e^{im\phi} \cos\left(kr - \frac{\pi}{2}\left(m + \frac{1}{2}\right)\right). \tag{35}$$

Expressing the spinors in Eq. (25) and in Eq. (34) in terms of incoming and outgoing waves, and equating the pre-factors corresponding to $e^{-ik_\perp r}$, we obtain a system of equations from which we determine the coefficient C_m. We obtain

$$C_m = \frac{i^m}{\sqrt{1+\beta^2}} e^{i\delta_m}. \tag{36}$$

Applying the same procedure for the pre-factors of $e^{ik_\perp r}$, we find that the scattering amplitude is

$$\begin{bmatrix} f_1(\phi) \\ f_2(\phi) \end{bmatrix} = \frac{e^{-\frac{i\pi}{4}}}{\sqrt{2\left(1+\beta^2\right)\pi k_\perp}} \sum_{m=-\infty}^{\infty} \begin{bmatrix} e^{im\phi} \\ \beta e^{i(m+1)\phi} \end{bmatrix} \left(e^{2i\delta_m} - 1\right). \tag{37}$$

Note that from Eq. (37) we have a relation between $f_1(\phi)$ and $f_2(\phi)$, namely

$$f_2(\phi) = \beta e^{i\phi} f_1(\phi). \tag{38}$$

Scattering Cross-Section

The differential cross-section (per unit of cylinder length) is given by the square modulus of the vector in Eq. (37), i.e.,

$$\frac{d}{d\phi}(\sigma/L) = |f_1(\phi)|^2 + |f_2(\phi)|^2 = \frac{2}{\pi k_\perp}\sum_{m,m'} e^{i(\delta_m - \delta_{m'})}\sin\delta_m \sin\delta_{m'} e^{i(m-m')\phi}, \tag{39}$$

and the total cross-section (per unit of cylinder length) is calculated from Eq. (39) by integrating over the scattering angle, $0 \leq \phi \leq 2\pi$. We get

$$\sigma/L = \frac{4}{k_\perp}\sum_{m=-\infty}^{\infty}\sin^2\delta_m. \tag{40}$$

3 Transmission and Landauer formalism

In this section we will study the transport properties of a slab made of a WSM of height L (z-direction) and width W (y-direction). We assume that $L, W \gg 1/k_F$. The slab is connected to two semi-infinite WSM contacts which are maintained at chemical potentials μ_L and μ_R, respectively. The particle flux (particles per unit time per unit area) emitted by the left/right contact is defined as

$$dJ_{L/R} = v_F \cos\phi D_{L/R}(E) f_{L/R}(E) dE, \tag{41}$$

where $D_{L/R}(E)$ is the density of states at the left/right contact and $f_{L/R}(E)$ is the equilibrium Fermi-Dirac distribution. In order to calculate the density of states, we assume that both contacts are identical semi-infinite regions of WSM, whose density of states are equal, and given by

$$D_L(E) = D_R(E) = D(E) = 4\int \frac{d^3k}{(2\pi)^3}\left[\delta\left(E - \hbar v_F k\right) + \delta\left(E + \hbar v_F k\right)\right], \tag{42}$$

where the factor of 4 arises from the spin and node degeneracy at each of the WSM semi-infinite contacts. Performing the integration we obtain

$$D(E) = \frac{2|E|}{\pi(\hbar v_F)^2 L}\theta(|E|). \tag{43}$$

From now on, we consider that the incident spinor has a wave vector $\mathbf{k} = (k_\perp, 0, 0)$, i.e. a plane wave propagating in the $x-$direction. The effect of the scattering center (the cylindrical strip) on the transport properties can be expressed as an effective cross-section $\sigma_{\text{eff}}(E)$. For this purpose we define the energy-

dependent transmission coefficient as

$$\bar{T}(E) = \int_{-\pi/2}^{\pi/2} d\phi \cos\phi \frac{1}{\sigma(E)} \frac{d\sigma}{d\phi}. \tag{44}$$

Here, $\sigma(E)$ is the total cross-section at energy E. In order to compute the angular average we need to use the following result

$$\int_{-\pi/2}^{\pi/2} d\phi \cos\phi \, e^{i(m-m')\phi} = \delta_{m-m',2p} \frac{2\,(-1)^{p+1}}{4p^2-1}, \;\; p \in \mathbb{Z}. \tag{45}$$

For the case when the only scattering mechanism present is the RDSP, we can write the effective cross-section as

$$\sigma_{\text{eff}}^{RD} = \pi a L \bar{T}(E_{\lambda,k_\perp}), \tag{46}$$

where $E_{\lambda,k_\perp}$ is the energy of the free pseudo-spinor with momentum $\mathbf{k}$. The particle flow (particles per unit time) along the x-direction emitted by the left/right contact, and arising from the $\mathbf{K}_\xi$ node can be written as $d\dot{N}^{\xi}_{L/R} = \sigma_{\text{eff}}(E)\, dJ_{L/R}$. The net electric current flowing across the region will be $I = I_+ + I_-$, with the node component given by

$$I_\xi = e \int \left(d\dot{N}^{\xi}_{x,L} - d\dot{N}^{\xi}_{x,R} \right). \tag{47}$$

Replacing Eq. (41) for the particle flux, the density of states Eq. (43), the effective cross-section Eq. (46), the transmission coefficient Eq. (44) in terms of the differential cross section Eq. (39), and after explicit angular integration using the result in Eq. (45) the total electric current is given by

$$I = 2 e v_F \sum_\lambda \int_0^\infty dk_\perp \mathcal{T}\left(E_{\lambda,k_\perp}\right) \left[f_L\left(E_{\lambda,k_\perp}\right) - f_R\left(E_{\lambda,k_\perp}\right) \right], \tag{48}$$

where the factor 2 arises from the symmetric contribution of the two chiral nodes, $\xi = \pm 1$. Here we use the symbol $\mathcal{T}(E)$ as a convenient shorthand notation for the transmission function

$$\mathcal{T}\left(E_{\lambda,k_\perp}\right) = \frac{8\xi a L}{\pi} \sum_{m,p} \frac{\lambda(-1)^{p+1}}{\sigma\left(E_{\lambda,k_\perp}\right)(4p^2-1)} e^{i(\delta_m - \delta_{m-2p})} \sin(\delta_m)\sin(\delta_{m-2p}). \tag{49}$$

It is important to notice that the phase shifts appearing in this expression are those given in Eq. (29). Now, we calculate the differential conductance $G(T,V) = \partial I/\partial V|_T$. For simplicity, we assume that $\mu_L = \mu_R + eV$ and $T_L = T_R = T$; we get finally

$$G(T,V) = 2\frac{e^2 v_F}{k_B T} \sum_\lambda \int_0^\infty dk_\perp \mathcal{T}(E_{\lambda,k_\perp}) f_L(E_{\lambda,k_\perp}) \left[1 - f_L(E_{\lambda,k_\perp})\right], \tag{50}$$

where we have used that

$$\frac{\partial f_L(E,V)}{\partial V} = \frac{e}{k_B T}\left[1 - f_L(E,V)\right] f_L(E,V). \tag{51}$$

On the other hand, when we have the combined effect of the external magnetic field and the torsion pseudo-field inside the cylinder, in addition to the RDSP at the boundary, the effective cross-section can be written in the form[3]

$$\sigma_{\text{eff}} = L \sum_{n,\lambda} \bar{T}(E^{\xi}_{\lambda,n}) \delta\left(\lambda k_{\perp} - \frac{E^{\xi}_{\lambda,n}}{\hbar v_F}\right), \tag{52}$$

where $E^{\xi}_{\lambda,n}$ is the pseudo-Landau energy spectrum inside the cylindrical region, and the Dirac delta function enforces the energy conservation condition assumed for elastic scattering. Proceeding as before but with the effective cross-section given in Eq. (52), the node component of the current is

$$I_{\xi} = e v_F \sum_{n,\lambda} \mathcal{T}(E^{\xi}_{\lambda,n}) \left[f_L(E^{\xi}_{\lambda,n}) - f_R(E^{\xi}_{\lambda,n}) \right], \tag{53}$$

where the effective transmission function, for this case, is

$$\mathcal{T}(E^{\xi}_{\lambda,n}) = \frac{8L}{\pi^2} \sum_{m,p} \frac{(-1)^{p+1}}{\sigma(E^{\xi}_{\lambda,n})(4p^2-1)} e^{i(\delta_m - \delta_{m-2p})} \sin\delta_m \sin\delta_{m-2p}, \tag{54}$$

and the phase shifts here are those in Eq. (32). The differential conductance for this case is

$$G(T,V) = \frac{e^2 v_F}{k_B T} \sum_{\lambda,n,\xi} \mathcal{T}(E^{\xi}_{\lambda,n}) f_L(E^{\xi}_{\lambda,n}) \left[1 - f_L(E^{\xi}_{\lambda,n}) \right]. \tag{55}$$

Finally, in the low temperature limit the Fermi distribution becomes a Heaviside function

$$f_L(E,V) = \Theta\left(\frac{\mu_R + eV - E}{k_B T}\right), \tag{56}$$

and the derivative in Eq.(51) becomes a delta function

$$\frac{\partial f_L(E,V)}{\partial V} = e\delta\left(E - \mu_R - eV\right). \tag{57}$$

Then, directly from Eq.(48) or Eq. (53), we have that the conductance at low temperatures can be approximated by

$$G(V) = e^2 v_F \mathcal{T}\left(eV + \mu_R\right) D\left(eV + \mu_R\right), \tag{58}$$

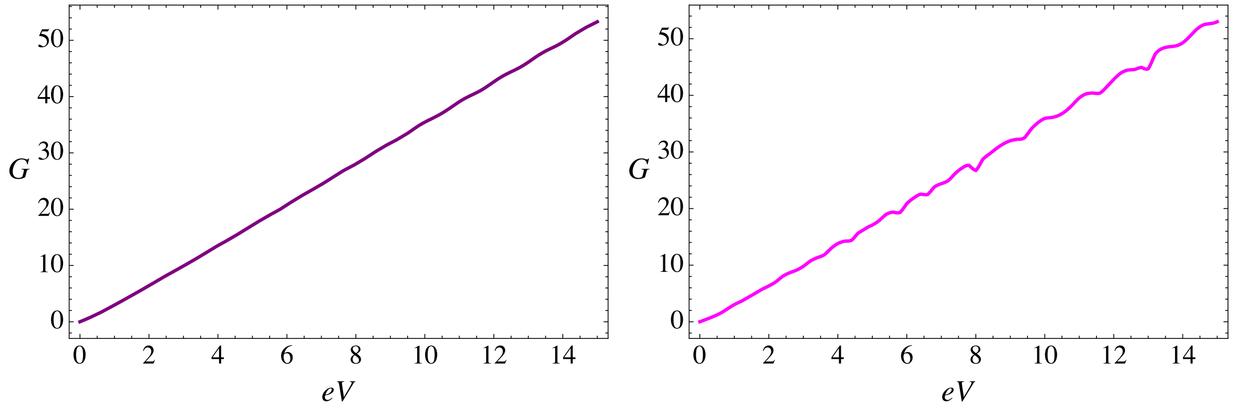

(a) (b)

Fig. 1: (Color online) Differential conductance (in units of $e^2/\hbar$) for the RDSP, plotted as function of applied bias eV (in units of $\hbar v_F/a$) for $\alpha = 3\pi/4$ and $T = 0.2\,\hbar v_F/k_B a$. The Subfig. (a) is computed from the analytical expression in Eq. (50) whereas the Subfig. (b) is computed from the low temperature expression in Eq. (58).

where $D(E)$ is the density of states; for the case of RDSP alone it is the free density of states in Eq. (43), whereas for the case of magnetic and torsion pseudo-fields inside the cylinder and RDSP at the boundary, it corresponds to the density of states for the pseudo-Landau energy spectrum. A comparison of the differential conductance for the case when the only scattering mechanism is the RDSP is presented in Fig. 1.

4 Thermoelectric transport coefficients

We start with the expression given in the text for the energy current accross the junction arising from each chiral node contribution $\xi = \pm$

$$\dot{U}_\xi = v_F \sum_{n,\lambda} E^\xi_{\lambda,n} \mathcal{T}(E^\xi_{\lambda,n}) \left[f_L(E^\xi_{\lambda,n}) - f_R(E^\xi_{\lambda,n}) \right]. \tag{59}$$

and the net heat current transmitted across the junction

$$\dot{Q}_\xi = \dot{U}_\xi - \left(\mu_L \dot{N}^\xi_L - \mu_R \dot{N}^\xi_R \right). \tag{60}$$

For the thermal conductance we have, as usual, its definition under conditions where there is no net electric current $(I = 0)$

$$\kappa(T, V) = - \left. \frac{\partial \dot{Q}}{\partial \Delta T} \right|_{I=0} = - \left. \frac{\partial \dot{U}}{\partial \Delta T} \right|_{I=0}, \tag{61}$$

where $\Delta T = T_R - T_L$. The condition of a vanishing electric current defines an implicit relation between the voltage difference and the thermal gradient across the junction, by $I(\Delta T, V, T) = 0$. At constant T, we have

$$dI(\Delta T, T, V) = \left.\frac{\partial I}{\partial \Delta T}\right|_{T,V} d\Delta T + \left.\frac{\partial I}{\partial V}\right|_{\Delta T, T} dV = 0, \tag{62}$$

then

$$\left.\frac{\partial I}{\partial \Delta T}\right|_{T,V} + \left.\frac{\partial I}{\partial V}\right|_{\Delta T,T} \left.\frac{\partial V}{\partial \Delta T}\right|_{I=0,T} = 0. \tag{63}$$

Finally, we have for the Seebeck coefficient

$$S(T,V) = -\left.\frac{\partial V}{\partial \Delta T}\right|_{I=0,T} = \frac{\left.\frac{\partial I}{\partial \Delta T}\right|_{T,V}}{\left.\frac{\partial I}{\partial V}\right|_{T,\Delta T}}, \tag{64}$$

where $\Delta T(V,T)$ is obtained as the solution of the equation $I(T,V,\Delta T) = 0$. Following the argument above, the thermal conductance defined in Eq. (61) is calculated by means of the chain rule in terms of the Seebeck coefficient

$$\kappa(T,V) = -\left.\frac{\partial \dot{U}}{\partial \Delta T}\right|_{T,V} + S(T,V)\left.\frac{\partial \dot{U}}{\partial V}\right|_{T,\Delta T}. \tag{65}$$

For simplicity, we assume that $T_L = T$, $T_R = T + \Delta T$, $\mu_R = \mu$ and $\mu_L = \mu + eV$. Then, we have for the derivatives of the Fermi distributions

$$\frac{\partial f_R(E)}{\partial \Delta T} = \frac{E - \mu}{k_B \left(T + \Delta T\right)^2}\left[1 - f_R(E)\right] f_R(E), \tag{66}$$

and the result in Eq. (51). Now, from Eq. (59) and Eq. (60), we obtain by explicit integration

$$\dot{Q}_\xi = v_F \sum_{n,\lambda} \mathcal{T}(E^\xi_{\lambda,n})\left[(E^\xi_{\lambda,n} - \mu_L) f_L(E^\xi_{\lambda,n}) - (E^\xi_{\lambda,n} - \mu_R) f_R(E^\xi_{\lambda,n})\right], \tag{67}$$

and

$$\dot{U}_\xi = v_F \sum_{n,\lambda} \mathcal{T}(E^\xi_{\lambda,n}) E^\xi_{\lambda,n}\left[f_L(E^\xi_{\lambda,n}) - f_R(E^\xi_{\lambda,n})\right], \tag{68}$$

where the effective transmission function, $\mathcal{T}(E^{\xi}_{\lambda,n})$, is given in Eq.(54). Now, from Eq.(53) and using the result in Eq.(66), we have

$$\left.\frac{\partial I}{\partial \Delta T}\right|_{T,V} = \sum_{\xi} \left.\frac{\partial I_{\xi}}{\partial \Delta T}\right|_{T,V} = -\frac{e v_F}{k_B (T+\Delta T)^2} \sum_{n,\lambda,\xi} \mathcal{T}(E^{\xi}_{\lambda,n}) \left(E^{\xi}_{\lambda,n} - \mu\right) f_R(E^{\xi}_{\lambda,n}) \left[1 - f_R(E^{\xi}_{\lambda,n})\right], \quad (69)$$

and from Eq.(55)

$$\left.\frac{\partial I}{\partial V}\right|_{T,\Delta T} = \frac{e^2 v_F}{k_B T} \sum_{\lambda,n,\xi} \mathcal{T}(E^{\xi}_{\lambda,n}) f_L(E^{\xi}_{\lambda,n}) \left[1 - f_L(E^{\xi}_{\lambda,n})\right]. \quad (70)$$

Thus, we obtain the explicit analytical expression for the Seebeck coefficient in Eq.(64)

$$S(T,V) = -\frac{T \sum_{\lambda,n,\xi} \mathcal{T}(E^{\xi}_{\lambda,n}) \left(E^{\xi}_{\lambda,n} - \mu\right) f_R(E^{\xi}_{\lambda,n}) \left[1 - f_R(E^{\xi}_{\lambda,n})\right]}{e (T+\Delta T)^2 \sum_{\lambda,n,\xi} \mathcal{T}(E^{\xi}_{\lambda,n}) f_L(E^{\xi}_{\lambda,n}) \left[1 - f_L(E^{\xi}_{\lambda,n})\right]}. \quad (71)$$

In order to compute the thermal conductance, we have from Eq.(68) and the result in Eq.(66)

$$\begin{aligned} \left.\frac{\partial \dot{U}}{\partial \Delta T}\right|_{T,V} &= \sum_{\xi} \left.\frac{\partial \dot{U}_{\xi}}{\partial \Delta T}\right|_{T,V} \\ &= -\frac{v_F}{k_B (T+\Delta T)^2} \sum_{\lambda,n,\xi} \mathcal{T}(E^{\xi}_{\lambda,n}) E^{\xi}_{\lambda,n} \left[E^{\xi}_{\lambda,n} - \mu\right] f_R(E^{\xi}_{\lambda,n}) \left[1 - f_R(E^{\xi}_{\lambda,n})\right], \quad (72) \end{aligned}$$

and using the result in Eq.(51)

$$\left.\frac{\partial \dot{U}}{\partial V}\right|_{T,\Delta T} = \sum_{\xi} \left.\frac{\partial \dot{U}_{\xi}}{\partial V}\right|_{T,\Delta T} = \frac{e v_F}{k_B T} \sum_{\lambda,n,\xi} \mathcal{T}(E^{\xi}_{\lambda,n}) E^{\xi}_{\lambda,n} f_L(E^{\xi}_{\lambda,n}) \left[1 - f_L(E^{\xi}_{\lambda,n})\right]. \quad (73)$$

Finally, the explicit analytical expression for the thermal conductance obtained from Eq.(65) is

$$\begin{aligned} \kappa(T,V) &= \frac{v_F}{k_B (T+\Delta T)^2} \sum_{\xi,\lambda,n} \mathcal{T}(E^{\xi}_{\lambda,n}) E^{\xi}_{\lambda,n} \left[E^{\xi}_{\lambda,n} - \mu\right] f_R(E^{\xi}_{\lambda,n}) \left[1 - f_R(E^{\xi}_{\lambda,n})\right] \\ &\quad + S(T,V) \frac{e v_F}{k_B T} \sum_{\lambda,n,\xi} \mathcal{T}(E^{\xi}_{\lambda,n}) E^{\xi}_{\lambda,n} f_L(E^{\xi}_{\lambda,n})) \left[1 - f_L(E^{\xi}_{\lambda,n})\right]. \quad (74) \end{aligned}$$

5 Estimation of the parameter V_0

Assuming a WSM wire of length L, with the axis along the z direction, the displacement vector in cylindrical coordinates is [5]

$$\mathbf{u} = \theta \frac{z}{L} (\mathbf{r} \times \hat{z}), \quad (75)$$

where θ is the torsion angle in radians, and $\mathbf{r}$ is the radial distance vector (in the $x-y$ plane) measured from the axis of the cylinder. The maximum displacement vector occurs at the radius of the cylinder $r=a$, and for $z=L$. Then, the magnitude of this vector is approximately (small angles)

$$|\mathbf{u}|_{\max} \approx a\theta. \tag{76}$$

The energy per unit length is relatively insensitive to the character of the dislocation (screw, edge, etc). This energy per unit length can be roughly written as [6]

$$\bar{E} \approx \gamma G u_{\max}^2 = \gamma G a^2 \theta^2, \tag{77}$$

where G is the 'shear modulus' (in units of pressure) and γ is a dimensionless factor between 0.5 and 1. This result is known as *Frank's rule.* In our case, the total energy of dislocation for a cylinder of radius a and axial length L is (we assume here that $\gamma = 1$)

$$E = G_H a^2 \theta^2 L. \tag{78}$$

Here $G_H = 91.88$ GPa for TaAs [7]. Because of the presence of $\delta(r-a)$, we have to multiply the energy in the Eq. (78) by the radial distance a. Then, a crude estimation of the value of V_0 would be

$$V_0 \approx G_H a^2 \theta^2 L \times a = a^3 \theta^2 G_H L. \tag{79}$$

We use the following reference values[8]: $a = 50$ nm, $L = 100$ nm, $\theta = 15^\circ = (15/180)\pi$. Then $V_0 = 4.91$ GeV Å. We know that $\hbar = 6.58 \times 10^{-16}$ eV s and the Fermi velocity for TaAs is 1.3×10^5 m s^{-1}, so that $\hbar v_F = 0.855$ eV Å. The value of α is

$$\alpha = \frac{V_0}{\hbar v_F} = \frac{4.91 \times 10^9 \text{ eV Å}}{0.855 \text{ eV Å}} = 5.74 \times 10^9. \tag{80}$$

Now, since $\tan \delta_m$ is a periodic function of α with period π, i.e. $\tan \delta_m(\alpha) = \tan \delta_m(\alpha + \pi)$, we define the effective value of $\alpha(\text{mod}\pi)$ by

$$\begin{aligned} \alpha(\text{mod}\pi) &= 5.74 \times 10^9 - \pi \cdot \lfloor 5.74 \times 10^9/\pi \rfloor = 2.183 \\ &\approx 0.695\pi. \end{aligned} \tag{81}$$

There are another estimations of G_H. For example, $G = 54$ GPa [9] gives a value $V_0 = 2.89$ GeV Å, and hence an effective value of $\alpha(\mathrm{mod}\pi)$

$$\begin{aligned}\alpha(\mathrm{mod}\pi) &= 2.89\times 10^9/0.855 - \pi\cdot\lfloor 2.89\times 10^9/0.855/\pi\rfloor = 2.023\\ &\approx 0.644\pi. \end{aligned} \tag{82}$$

Therefore, for the sake of the examples presented in this work, we choose $\alpha = 3\pi/4$ as a representative value.

References

[1] Jun John Sakurai. *Modern Quantum Mechanics; rev. ed.* Addison-Wesley, Reading, MA, 1994.

[2] Enrique Muñoz and Rodrigo Soto-Garrido. Analytic approach to magneto-strain tuning of electronic transport through a graphene nanobubble: perspectives for a strain sensor. *Journal of Physics: Condensed Matter*, 29(44):445302, 2017.

[3] Rodrigo Soto-Garrido and Enrique Muñoz. Electronic transport in torsional strained Weyl semimetals. *Journal of Physics: Condensed Matter*, 30(19):195302, 2018.

[4] A. Jeffrey. Editor's foreword. In I.S. Gradshteyn and I.M. Ryzhik, editors, *Table of Integrals, Series, and Products.* Academic Press, 1980.

[5] Vicente Arjona and María A. H. Vozmediano. Rotational strain in Weyl semimetals: A continuum approach. *Physical Review B*, 97:201404, 2018.

[6] D. Hull and D.J. Bacon. Chapter 4 - elastic properties of dislocations. In D. Hull and D.J. Bacon, editors, *Introduction to Dislocations (Fifth Edition)*, pages 63–83. Butterworth-Heinemann, Oxford, fifth edition edition, 2011.

[7] M. I. Naher and S. H. Naqib. An ab-initio study on structural, elastic, electronic, bonding, thermal, and optical properties of topological Weyl semimetal TaX (X = P, As). *Scientific Reports*, 11(1):5592, 2021.

[8] D. I. Pikulin, Anffany Chen, and M. Franz. Chiral Anomaly from Strain-Induced Gauge Fields in Dirac and Weyl Semimetals. *Physical Review X*, 6:041021, 2016.

[9] Lei Liu, Zhao-Qi Wang, Cui-E. Hu, Yan Cheng, and Guang-Fu Ji. Comparative study on structural, elastic, dynamical, and thermodynamic properties of Weyl semimetals MX (M = Ta or Nb; X = As or P). *Solid State Communications*, 263:10–18, 2017.
